# Validation of the Sysmex XN-V Automated Nucleated Red Blood Cell Enumeration for Canine and Feline EDTA-Anticoagulated Blood

**DOI:** 10.3390/ani14030455

**Published:** 2024-01-30

**Authors:** Julia Ginders, Martina Stirn, Marilisa Novacco, Regina Hofmann-Lehmann, Barbara Riond

**Affiliations:** Clinical Laboratory, Department of Clinical Diagnostics and Services, Vetsuisse Faculty, University of Zurich, Winterthurerstrasse 260, 8057 Zurich, Switzerland; martina.stirn@roche.com (M.S.); mnovacco@vetclinics.uzh.ch (M.N.); rhofmann@vetclinics.uzh.ch (R.H.-L.); briond@vetclinics.uzh.ch (B.R.)

**Keywords:** veterinary hematology, metarubricytosis, rubricytosis, normoblastosis, dogs, cats, precision, method comparison, manual count, nRBC

## Abstract

**Simple Summary:**

The presence of nucleated red blood cells in the peripheral blood (normoblastosis) of dogs and cats can be associated with different diseases and represents a negative prognostic factor in critically ill patients. Most veterinary automated hematological analyzers do not provide an enumeration of nucleated red blood cells, counting them instead as white blood cells. This makes a blood smear evaluation essential for the detection of nucleated red blood cells and subsequent correction of the automated white blood cell count. However, manual counts are known to be time-consuming and imprecise, and cases with normoblastosis may remain undetected if blood smear evaluation is not routinely performed. As the first veterinary hematology analyzer, Sysmex XN-V provides an automated nucleated red blood cell count. By comparing the results of manual and automated counts of 3810 canine and 2844 feline samples, this study demonstrates that the automated nucleated red blood cell count performed by the Sysmex XN-V hematology analyzer is accurate and can replace manual counts. Additionally, automated counts show a lower imprecision compared to manual counts. The automated nucleated red blood cell count not only represents a time-saving and cost-efficient advancement but also adds value to automated veterinary hematology profiles.

**Abstract:**

The enumeration of nRBCs (nucleated red blood cells) by manual counting is time-consuming and imprecise. As the first veterinary hematology analyzer, Sysmex XN-V provides automated nRBC counts. This study aimed to evaluate the performance of Sysmex XN-V in the enumeration of nRBCs for cats and dogs by comparing automated nRBC counts to manual counts from a total of 3810 canine and 2844 feline specimens. Repeatability, reproducibility, stability, carry-over, and linearity were assessed. The repeatability and reproducibility of Sysmex XN-V were good, with mean coefficients of variation (CV) of 4.5% and 5.4%, respectively. Bland–Altman difference analysis revealed mean biases shown as nRBCs/100 WBCs of 0.01 in dogs and 0.11 in cats with low nRBCs (<5/100 WBCs), mean biases of −1.27 in dogs and −0.24 in cats with moderate nRBC counts (5–20 nRBCs/100 WBCs), and mean biases of −7.76 in dogs and −1.31 in cats with high nRBC counts (>20 nRBCs/100 WBCs). The total observable error was below 9% in both species and at all ranges. Overall concordance between methods was high (91% in canine and 93% in feline samples). The automated nRBC count by Sysmex XN-V was found to be accurate and precise and can replace manual counts for cat and dog samples. Non-statistical quality assurance by scattergram evaluation, re-gating, and confirmation by blood smear evaluation is, however, recommended, especially in cases with severe normoblastosis. This advancement will save time, reduce errors, and add prognostic value to hematological results for animal patients.

## 1. Introduction

Nucleated red blood cells (nRBCs) are usually absent in the peripheral blood of cats and dogs, as erythropoiesis mostly occurs in the bone marrow. During erythropoiesis, hematopoietic stem cells proliferate under the influence of growth and transcription factors, resulting in a continuous mitotic division from rubriblasts to prorubricytes, basophilic rubricytes, and polychromatophilic rubricytes. Polychromatophilic rubricytes are post-mitotic and undergo maturation to metarubricytes before extruding their nucleus and developing into reticulocytes [1]. In dogs and cats, reticulocytes mature partially still within the bone marrow and also after their release into the peripheral bloodstream, preferentially in the spleen, where hemoglobinization, membrane remodeling, and cytoskeletal stabilization are completed [2]. In the case of the release of erythroid precursors from bone marrow, splenic macrophages also contribute to the quick removal of nuclei, nuclear remnants, and organelles. The exact mechanism that prevents nRBCs from being released into peripheral blood in health is not yet fully understood. It is suspected that the adhesion properties of specialized reticular cells of the blood–bone marrow barrier and the rigidity of nRBCs may prevent nRBCs from being released [3].

The presence of nRBCs in the peripheral blood, independent of their maturation stage, is commonly termed normoblastosis (term used for this publication) or normoblastemia. Physiological normoblastosis may be seen in response to severe regenerative anemia (appropriate normoblastosis), e.g., secondary to blood loss or immune-mediated hemolytic anemia. Consequently, the nRBCs are accompanied by reticulocytosis, which can be appreciated as polychromasia and anisocytosis upon blood smear evaluation. Inappropriate normoblastosis in dogs and cats is commonly associated with different pathological processes such as lead toxicity, heatstroke, splenic disorders, or bone marrow injury secondary to, e.g., neoplasia, hypoxia, septicemia, or drug administration [4,5,6,7,8]. Additionally, inappropriate normoblastosis is described in congenital dyserythropoiesis in English Springer Spaniel and familial Poodle macrocytosis [9]. Mild normoblastosis is also reported in dogs and cats suffering from cardiovascular disease, respiratory disease, trauma, inflammatory conditions, or hyperadrenocorticism, and extramedullary hematopoiesis may also contribute to normoblastosis [10].

In recent publications, normoblastosis has been examined as a potential prognostic biomarker involving canine and feline patients. In veterinary critical care, novel, readily available, and cost-efficient prognostic biomarkers are continuously investigated to further improve the assessment of morbidity and mortality, as well as to monitor treatment response or disease progression. One study showed that normoblastosis in cats can be associated with more severe acute clinical signs, longer hospitalization, higher treatment costs, and lower survival rates and can therefore alert the clinician to the need to implement intensive care and add additional information regarding the prognosis of the patient [11]. In dogs, normoblastosis is most often associated with regenerative anemia and has been shown to be a negative prognostic indicator in these patients [12,13]. An association between normoblastosis and increased mortality has also been found in dogs with systemic inflammatory response syndrome (SIRS) after the exclusion of patients suffering from additional diseases commonly causing normoblastosis (e.g., hemolysis or hemorrhage) [14]. In dogs suffering from heatstroke, normoblastosis has been found to be a sensitive and specific predictor of death and secondary complications. Therefore, the enumeration of peripheral nRBCs has been suggested to be a useful additional analyte in the prediction of the outcome in dogs affected by heatstroke [15].

In the past, the automated enumeration of nRBCs was unavailable in veterinary medicine, and microscopical enumeration by blood smear evaluation is still most often used for the enumeration of nRBCs. Although simple, the manual method is time-consuming, known to be imprecise, and may miss low numbers of nRBCs [16,17]. Most available veterinary automated hematology analyzers do not provide an enumeration of nRBCs, thus counting nRBCs within the white blood cell (WBC) count. This necessitates an assessment of possible normoblastosis by blood smear evaluation with subsequent correction of automated WBC counts by the manually determined nRBC counts/100 WBCs. The equation used for the correction of WBC counts is as follows: corrected WBC (10^3^/µL) = (measured WBC cell count × 100)/(nRBC + 100) [18]. This previously unavoidable practice results in increased hands-on time and risk of error. Additionally, the failure to recognize significant normoblastosis can subsequently lead to erroneously high WBC counts, which may affect clinical decisions and the outcome of these patients [4,19].

A fast, accurate, and precise determination of nRBCs would represent an important advancement in automated veterinary hematology, add prognostic value to hematology profiles, save time, and reduce the risk of unnoticed normoblastosis. The Sysmex XN-V automated hematology analyzer is the first veterinary hematology analyzer providing automated nRBC counts alongside WBC counts for different species. The authors aimed to evaluate the automated nRBC enumeration performance of the Sysmex XN-V hematology analyzer for canine and feline patients, including method comparison, repeatability, reproducibility, linearity, stability, and carry-over.

## 2. Materials and Methods

### 2.1. Selection of Specimens

For method comparison, the study included canine and feline K3-ethylenediamine tetra-acetic acid (EDTA) whole blood specimens sent for routine diagnostic analysis to the Clinical Laboratory of the Vetsuisse Faculty, University of Zurich, Switzerland, between August 2019 and June 2022. All samples with an automated and manual nRBC count available were included in the study. Data analysis was performed retrospectively. The blood collection procedure was not standardized, and patients were included regardless of age, sex, breed, or disease. Most (>97%) specimens were collected in-house at the Small Animal Clinic of the Vetsuisse Faculty (K3-EDTA whole blood in MiniCollect, Greiner Bio-One tubes, Kremsmünster, Austria) and were readily submitted at room temperature. Few specimens (<3%) were collected at privately owned clinics in Switzerland and readily submitted by mail at ambient temperature, together with freshly prepared blood smears. After arrival at the Clinical Laboratory, all specimens were kept at room temperature, placed on a rotator (Labinco LD-76; Labinco, Breeda, The Netherlands) for at least five minutes, and gently homogenized manually before processing.

### 2.2. Manual Count by Blood Smear Evaluation

Blood smear preparation of in-house submitted samples was performed within one hour after sample collection by trained laboratory technicians. The smears were air-dried for 20 min before staining. Staining was performed on a HEMA-TEK 2000 slide stainer (Siemens Healthcare GmbH, Erlangen, Germany) with modified Wright’s stain. Experienced laboratory technicians counted the nRBCs by microscopic blood smear evaluation using a Leica DM LB2 microscope (Leica Microsystems AG, Heerbrugg, Switzerland) with 50× and 100× oil immersion objectives. The nRBCs were counted alongside the WBC differentiation (100 WBC differential). In cases of a left shift (presence of band neutrophils), a second 100 WBC count was performed by a second laboratory technician, and the means were calculated. This procedure was performed according to the laboratory’s non-statistical quality assurance and standard operating procedures to increase the precision of the manual differential. Manual nRBC counts were reported as nRBCs per 100 WBCs (nRBCm).

### 2.3. Automated Count by Sysmex XN-V

**Figure 1:**
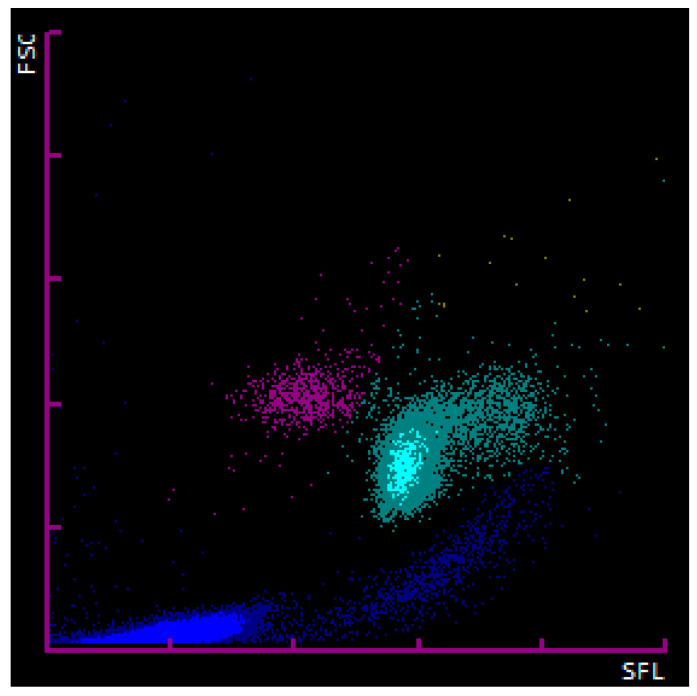
Graphic depiction (WNR-scattergram) of the flow cytometric analysis from a K3-ethylenediamine tetra-acetic acid (EDTA)-anticoagulated canine blood sample analyzed by the Sysmex XN-V automated hematological analyzer. The *X*-axis shows the side fluorescence light (SFL; proportional to the nucleic acid content) and the *Y*-axis shows the forward scattered light (FSC; proportional to the size) of the acquired cells. Each acquired cell is shown as a dot on the graph. Nucleated red blood cells (pink dots) appear with similar to slightly higher FSC and lower SFL compared to white blood cells (light blue). The nRBC and WBC populations appear well separated and were correctly assigned by the analyzer. Debris, platelets, and platelet aggregates are shown in dark blue. Basophils are shown in yellow.

Automated enumeration of the nRBCs was performed on the Sysmex XN-1000V analyzer (Sysmex Corporation, Kobe, Japan, Version 3.03 (00-08)) according to the manufacturer’s instructions using the species-specific preinstalled profiles for cats and dogs. In-house collected samples were processed within one hour after collection. The nRBCs were counted alongside the WBCs in the WNR (WBC/basophil/nRBC) channel of the analyzer. During the process, red blood cell (RBC) membranes are initially lysed by the hemolytic Lysercell WNR reagent. This reagent also shrinks WBCs while permeating their membrane, except for the basophil’s membrane, which, in certain species (humans and rabbits), remains relatively unaffected. As RBCs and nRBCs are the most affected by Lysercell WNR treatment, further RNA and nuclear degradation is suspected in nRBCs compared to WBCs. After lysis, nucleic acids are stained using Fluorocell WNR reagent. Subsequently, the forward scattered light (FSC) and side fluorescence light (SFL) properties of the cells are determined by flow cytometric analysis. The FSC and SFL properties display the size and nucleic acid content of the cells, respectively. This allows the analyzer to discriminate nRBCs from WBCs since nRBCs show less nucleic acid content (weak SFL) and are similar to those of a slightly larger size (medium FSC) compared to WBCs. The causes for nRBCs showing lower nucleic acid content (weaker SFL) are believed to be due to the loss of intracytoplasmic organelles/nucleic acids, smaller, more condensed nuclei, and RNA degradation by the Lysercell WNR reagent affecting the erythroid lineage to a higher extent than leukocytes [20]. An illustration of all of the acquired events is generated and shown as the associated WNR scattergram (FSC—*Y*-axis; SFL—*X*-axis) for each patient (Figure 1). At the Clinical Laboratory of the Vetsuisse Faculty Zurich, all of the WBC and nRBC counts were further investigated by performing an inspection for flags and evaluation of the associated scattergrams by experienced laboratory technicians. In the case of uncertain results, a blood smear evaluation was performed, and a clinical pathologist’s review was additionally requested. The nRBCs were measured as absolute counts (nRBCs/µL), and relative counts (nRBCs/100 WBCs; nRBCa) were calculated.

### 2.4. Method Comparison

For method comparison (determination of agreement), the relative nRBC counts established by Sysmex XN-V (nRBCa) were compared to the manually determined nRBC counts (nRBCm) using a total of 2844 feline and 3810 canine samples. In cases of significant differences between the automated and manual analysis (outliers), blood smears and automated data, including scattergrams, were revised and re-gating or manual re-counting was performed if necessary. For a few of the outliers, where a review of slides and/or automated data and scattergrams was not possible (e.g., due to missing slides), the data were dismissed (dogs, *n* = 3; cats, *n* = 1). The results were subdivided into three groups depending on the number of nRBCs/severity of normoblastosis determined by the manual method (low: <5; moderate: 5–20; high: >20 nRBCs/100 WBCs) to provide proper insight into the performance of different levels of normoblastosis.

### 2.5. Repeatability

The precision (repeatability) of the Sysmex XN-V automated count was evaluated from five consecutive measurements of three canine and two feline specimens. Additionally, manual precision was determined by five consecutive counts (nRBCs/100 WBCs) from four canine and four feline samples using blood smear evaluation. The counts were performed by the same observer (first author) on the same slide using an Olympus BX43 microscope (Evident Europe GmbH, Hamburg, Germany) with 50× and 100× oil immersion objectives.

### 2.6. Reproducibility

The precision from day to day (reproducibility) of Sysmex XN-V was assessed by using the data from the manufacturer’s control material of three different levels (XN-Check QC1 Lot. QC-23121101, QC2 Lot. QC-23121102, and QC3 Lot. QC-23121103; Sysmex Europe GmbH, Norderstedt, Germany), which were measured on 20 consecutive days. Quality control material was analyzed daily before analyzing the patient samples.

### 2.7. Linearity

The linearity was evaluated on Sysmex XN-V by using a dilution series (100%, 75%, 50%, 25%, and 0%) of fresh EDTA-anticoagulated blood diluted by isotonic saline (NaCl, sodium chloride 0.9%, Bichsel AG, Interlaken, Switzerland). Each level was measured in duplicate.

### 2.8. Stability

The stability (cell aging) was determined using two canine and two feline samples stored at either room temperature or refrigerated (4 °C). For both temperature levels, the samples were analyzed immediately after collection and 4, 8, 24, and 48 h later using the Sysmex XN-V.

### 2.9. Carry-Over

The carry-over was evaluated using two consecutive measurements of the patients’ EDTA-anticoagulated blood followed by three measurements of the blank (NaCl, sodium chloride 0.9%, Bichsel AG, Interlaken, Switzerland). Thereby, two canine EDTA samples with moderate (10.6 nRBCs/100 WBCs; 530 nRBCs/µL and) and high (42 nRBCs/100 WBCs; 9270 nRBCs/µL) nRBC counts were analyzed using the Sysmex XN-V.

### 2.10. Statistical Analysis

Data were collected in a Microsoft^®^ Excel^®^ spreadsheet (Microsoft 365 MSO Version 2306 Build 16.0.16529.20100, Microsoft Corp., Redmond, WA, USA) and statistical analysis was performed using the Analyse-it^®^ 6.01.1 add-in (Analyse-it Software Ltd., Leeds, UK). For precision analysis, the standard deviation (SD) and coefficient of variation (CV) were calculated for each level of nRBCs and species. The percentage of carry-over was determined by using the following formula: % carry-over = {[(result empty cycle 1) − (result empty cycle 3)]/[(sample 2) − (result empty cycle 3)]} × 100. Linearity was determined according to Emancipator–Kroll [21] using Analyse-it^®^. Stability was reviewed for statistical significance using a paired comparison test and the Friedman test (Analyse-it^®^). For method comparison (agreement), a Pearson coefficient of correlation (*r*), Deming linear regression analysis determining the intercept and the slope with a 95% confidence interval, and Bland–Altmann difference plot providing information on bias and the 95% limits of agreement were calculated for both species. The concordance of nRBC counts from the two assessed methods was analyzed using Microsoft^®^ Excel^®^ at four levels (≤1; 1.1–5; 5.1–20; >20 nRBCs/100 WBCs). The total observed error was calculated according to the ASVCP (American Society for Veterinary Clinical Pathology) guidelines [22]. The coefficient of correlation was considered excellent if *r* ≥ 0.95, very good if *r* = 0.90–0.94, good if *r* = 0.80–0.89, fair if *r* = 0.59–0.79, and poor if *r* < 0.59 [23]. Statistical significance was defined at a *p*-value of <0.05.

## 3. Results

### 3.1. Repeatability

The imprecision of manual counts performed on four canine samples with mean nRBC counts ranging from 5.2 to 58 nRBCs/100 WBCs showed CVs between 18.6% and 28.5%. The manual imprecision performed on four feline samples with mean nRBCs/100 WBCs ranging from 10 to 217 revealed CVs between 9.4% and 25.5%. The highest CVs were noted in the samples with the lowest nRBC counts in both species. The mean CV of the manual counts in the canine and feline specimens was 19.6%. The data are shown in Table 1.

The within-run imprecision (repeatability) of Sysmex XN-V using canine samples with low (330/µL), moderate (960/µL), and high (2998/µL) mean absolute nRBC counts showed a CV of 7.4%, 3.1%, and 2.3%, respectively. The within-run imprecision of Sysmex XN-V using two feline samples (268/µL and 398/µL) showed a CV of 3.1% and 6.5%, respectively. The mean CV for the within-run imprecision of Sysmex XV-V for the canine and feline specimens is 4.5%. The data are shown in Table 2.

### 3.2. Reproducibility

The quality control materials with mean absolute nRBC counts of 148, 473, and 1116 nRBCs/µL were analyzed. The between-run imprecision of Sysmex XN-V revealed a CV of 7.9%, 4.8%, and 3.5% for the Sysmex XN-V quality control materials QC1, QC2, and QC3, respectively. The mean CV was 5.4%. The data are shown in Table 2.

### 3.3. Method Comparison

For the analysis of agreement, the results were subdivided into three groups depending on the severity of normoblastosis determined by manual enumeration. For each group and species, coefficient of correlation (*r*), intercept and slope with 95% confidence intervals (CIs) calculated by Deming regression analysis, and biases with 95% limits of agreement (LoA) calculated by Bland–Altman difference plots are shown.

#### 3.3.1. Method Comparison of the Canine Samples

Manual nRBC counts in the canine samples (*n* = 3810) ranged from 0 to 272 nRBCs/100 WBCs. Most specimens (3584/3810; 94%) were found to have less than 5 nRBCs/100 WBCs, 4.6% (174/3810) were found with 5–20 nRBCs/100 WBCs, and 1.4% (52/3810) of samples showed more than 20 nRBCs/100 WBCs. The coefficient of correlation (*r*) of the three groups ranged from 0.754 to 0.978, indicating a fair correlation between manual and automated nRBC counts for the groups with less than 5 and 5–20 nRBCs/100 WBCs and excellent correlation for samples with more than 20 nRBCs/100 WBCs (Table 3). Deming regression analysis revealed an intercept of −0.10, −4.75, and 0.73 and a slope of 1.27, 1.37, and 0.82 for the low, moderate, and high groups, respectively. The 95% CI for the intercept did not include 0 in the two groups with ≤20 nRBCs/100 WBCs, indicating a constant difference between the two methods at the lower ranges of nRBC counts. The 95% CI for the slope did not include 1 in any of the groups, indicating a proportional difference between the two methods. Analysis of all canine samples as one group revealed a coefficient of correlation of 0.98, an intercept of 0.08 (95% CI: 0.02 to 0.15), and a slope of 0.84 (95% CI: 0.78 to 0.9), confirming the presence of a constant and proportional bias.

The distribution of nRBC counts determined by the manual method and Sysmex XN-V at different ranges (≤1, 1.1–5, 5.1–20, and >20 nRBCs/100 WBCs) are shown in Table 4. The overall concordance in all groups was high (91%; 3477/3810). The concordance in the group with ≤1 nRBCs/100 WBCs was the highest (96%; 3049/3188).

**Figure 2:**
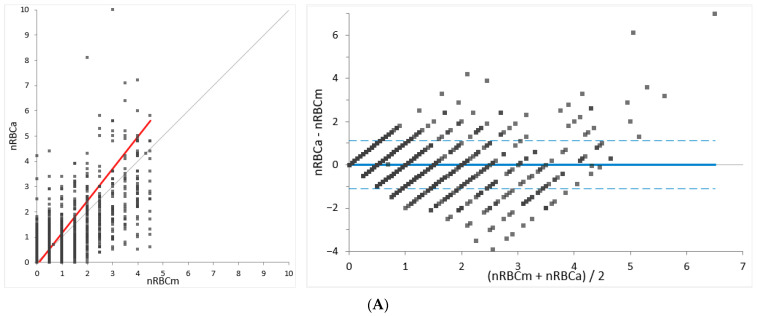

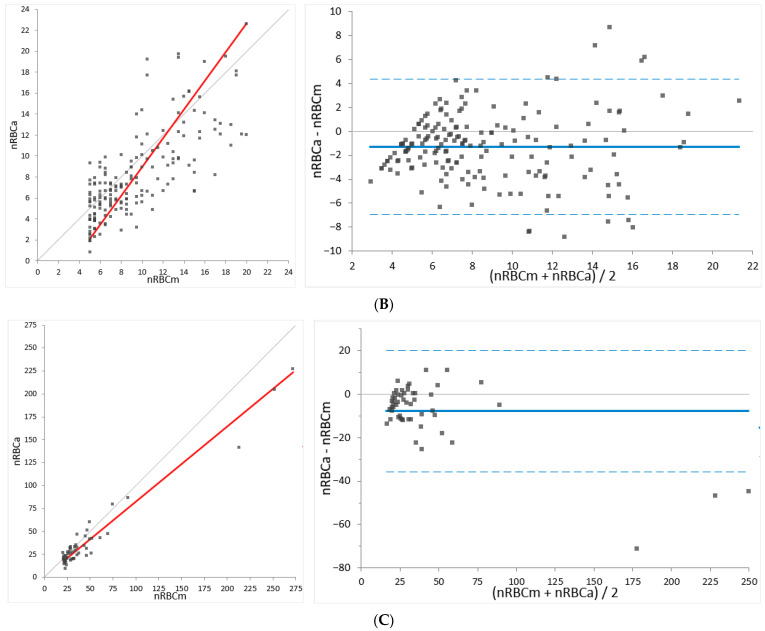
Deming regression (**left**) and Bland–Altman difference plot (**right**) for agreement analysis of the canine samples. Comparison of Sysmex XN-V relative nucleated red blood cell (nRBCa) counts and manually determined nRBCs/100 white blood cells (WBCs) (nRBCm). The canine specimens were divided into subgroups with (**A**) <5 nRBCs/100 WBCs, (**B**) 5–20 nRBCs/100 WBCs, and (**C**) >20 nRBCs/100 WBCs. The thin gray line in the Deming regression plots represents the line of identity (y = x) and the thick red line represents the line of best fit. In the Bland–Altman difference plots, the thin gray line at 0 of the *Y*-axis indicates the line of identity and the thick blue line indicates the mean difference between methods (mean bias is shown as nRBCs/100 WBCs). Lower and upper 95% limits of agreement (LoA) are shown as blue dashed lines.

Bland–Altman difference analysis revealed a mean bias of 0.01 nRBCs/100 WBCs in the group with low nRBCs (<5/100 WBCs), a mean bias of −1.27 nRBCs/100 WBCs in the group with moderate nRBCs (5–20 nRBCs/100 WBCs), and a mean bias of −7.76 nRBCs/100 WBCs in the group with high nRBCs (>20 nRBCs/WBCs). Narrow 95% limits of agreement (LoA) are noted for the group with low nRBC counts. In the groups with moderate and high nRBCs, the 95% LoA was wider (Table 3 and Figure 2). Bias among all samples was −0.16 nRBCs/100 WBCs (95% LoA: −4.2 to 3.9) (not graphically shown). The total observable error (TEobs = bias + 2 SD) for the canine samples ranged from 0.7% for samples with low nRBC counts (<5 nRBCs/100 WBCs) to 8.9% for samples with higher nRBCs/100 WBCs.

#### 3.3.2. Method Comparison of the Feline Samples

A total of 2844 feline samples were included in the method comparison study. Manual nRBC counts (nRBCm) ranged from 0 to 527 nRBCs/100 WBCs. Most specimens (2750/2844; 96.7%) revealed manual nRBC counts below 5/100 WBCs. Seventy-six (76/2844; 2.7%) feline samples were found to have 5–20 nRBCs/100 WBCs. Eighteen feline samples (18/2844; 0.6%) showed high nRBC counts (>20/100 WBCs). The coefficient of correlation (*r*) of the three groups ranged from 0.743 to 0.998, indicating fair correlation for the groups with less than 5 and 5–20 nRBCs/100 WBCs and excellent correlation for samples with more than 20 nRBCs/100 WBCs (Table 5). Deming regression analysis revealed an intercept of −0.04, −6.67, and −4.28 and a slope of 1.53, 1.66, and 1.04 for the low, moderate, and high groups, respectively. The 95% CI for the intercept did not include 0 in the two groups with ≤20 nRBCs/100 WBCs, indicating a constant difference between the two methods at the lower ranges of nRBC counts. The 95% CI for the slope did not include 1 in the two groups with ≤20 nRBCs/100 WBCs, indicating a proportional difference between the two methods. The group with nRBC counts above 20 showed no constant or proportional differences. Analysis of all feline samples as one group revealed a coefficient of correlation of 0.996, an intercept of 0.06 (95% CI: 0.02–0.1), and a slope of 1.03 (95% CI: 1–1.06), showing a constant but no proportional difference.

The distributions of feline nRBC counts determined by the manual method and Sysmex XN-V at different ranges (≤1, 1.1–5, 5.1–20, and >20 nRBCs/100 WBCs) are shown in Table 6. The overall concordance in all groups was high (93%; 2636/2844). Concordance in the group with ≤1 nRBCs/100 WBCs was the highest (95%; 2421/2844).

**Figure 3:**
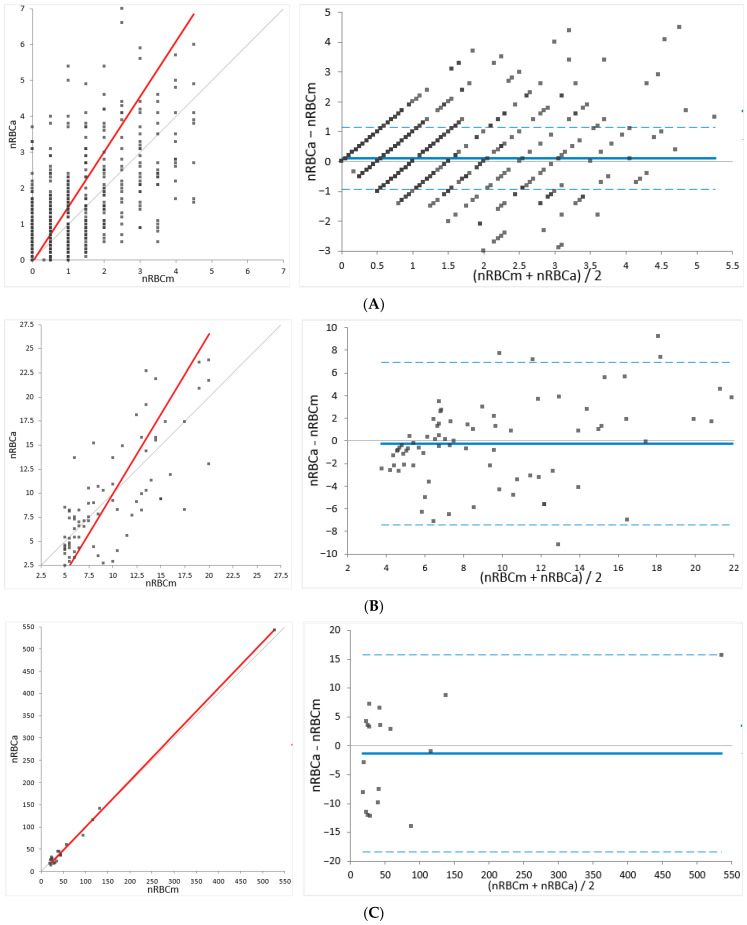
Deming regression (**left**) and Bland–Altman difference plot (**right**) for agreement analysis in the feline specimens. Comparison of Sysmex XN-V relative nucleated red blood cell (nRBCa) counts per 100 white blood cells (WBCs) and manually determined nRBCs/100 WBCs (nRBCm). The feline specimens are divided into subgroups with (**A**) less than 5 nRBCs/100 WBCs, (**B**) 5–20 nRBCs/100 WBCs, and (**C**) >20 nRBCs/100 WBCs. In the Deming regression plots, the thin gray line represents the line of identity (y = x) and the thick red line represents the line of best fit. In the Bland–Altman difference plots, the thin gray line at 0 of the *Y*-axis indicates the line of identity and the thick blue line indicates the mean difference between methods (the bias is determined as nRBCs/100 WBCs). The lower and upper 95% limits of agreement (LoA) are shown as blue dashed lines.

Bland–Altman difference analysis revealed a mean bias of 0.11 nRBCs/100 WBCs in the group with low nRBCs (<5/100 WBCs), a mean bias of −0.24 nRBCs/100 WBCs in the group with moderate nRBCs (5–20 nRBCs/100 WBCs), and a mean bias of −1.31 nRBCs/100 WBCs in the group with high nRBCs (>20 nRBCs/WBCs). Narrow 95% limits of agreement (LoA) are noted for the group with low nRBC counts. In the groups with moderate and high nRBCs, the 95% LoA was wider (Figure 3). The bias among all samples was 0.09 nRBCs/100 WBCs (95% LoA: −2 to 2.15) (not graphically shown). The TEobs for the feline samples ranged from 0.3% for samples with low nRBC counts (<5 nRBCs/100 WBCs) to 3% for samples with higher nRBCs/100 WBCs.

### 3.4. Linearity

**Figure 4:**
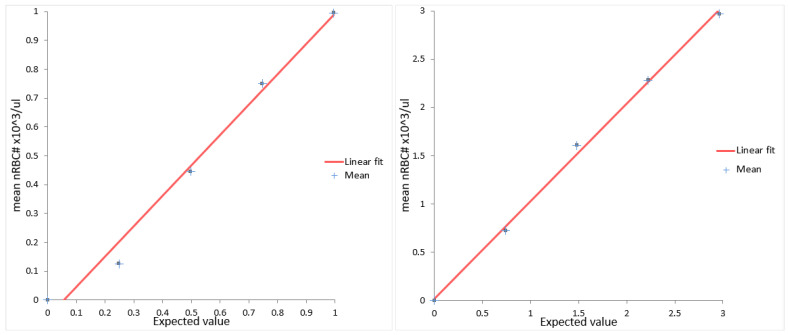
Linearity measurements of two canine samples at five levels (100%, 75%, 50%, 25%, and 0%). All samples were measured in duplicate, and the mean absolute nRBC values were plotted. nRBC = nucleated red blood cell.

For linearity testing, two canine samples with 2965 and 995 nRBCs/µL were assessed in duplicate. The maximum difference from linearity was shown at the lowest measurement (25% of 955 nRBCs/µL) with an absolute difference of −114 nRBCs/µL and a relative difference of −91%. The relative difference of −91% exceeded the limit for the difference in linearity for absolute nRBC counts reported by the manufacturer (±10% or ±200 nRBCs/µL). All other measurements showed a small difference in linearity, below 8% (Figure 4).

### 3.5. Stability

The results from the stability study performed on Sysmex XN-V are shown in Table 7. No statistically significant changes were observed for either species or temperature levels. In the case of one cat, the measurement at 48 h could not be determined, due to insufficient sample material.

### 3.6. Carry-Over

None of the measured blank samples showed nRBCs, revealing no detectable carry-over in either of the tested samples (530 and 9270 nRBCs/µL).

### 3.7. Case Demonstration of the Severe Error of Automated nRBC Counts

**Figure 5:**
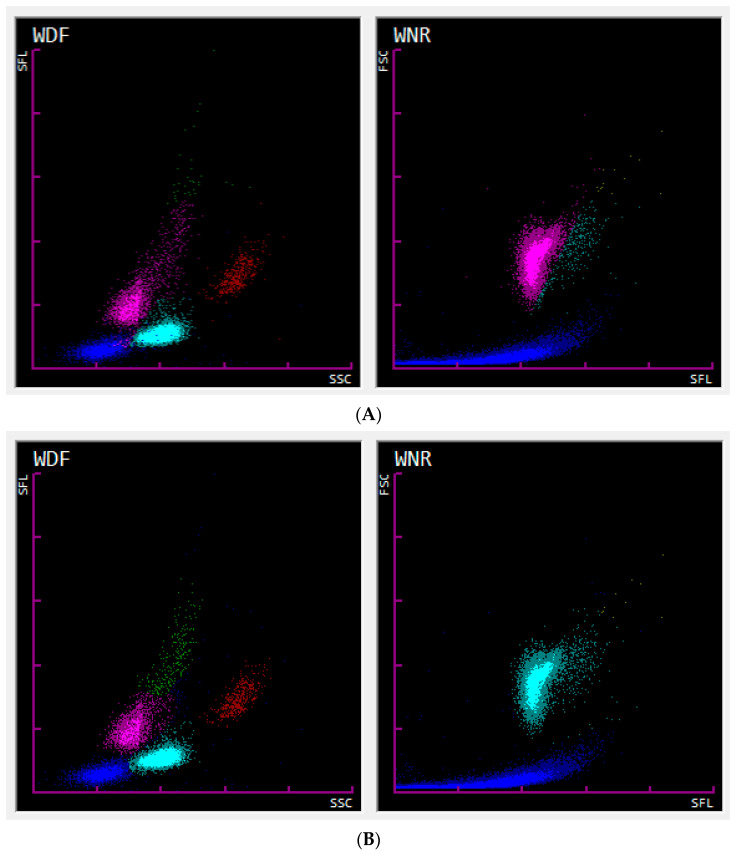
WDF (**left**) and WNR (**right**) scattergrams from Cat 51. The corresponding data are shown in Table 8. (**A**) Sysmex XN-V scattergrams before manual re-gating. A misclassification of WBCs as nRBCs is seen in the WNR channel. The WDF scattergram shows normal distribution and fractioning of cell populations. (**B**) Scattergrams after manual re-gating to match the manually determined nRBC count (nRBCm = 0.5 nRBCs/100 WBCs). WNR and WDF scattergrams show a normal distribution and fraction of cell populations. Abbreviations: SSC = side scattered light (proportional to the granularity of the cell); FSC = forward scattered light (proportional to the cell size); SFL = side fluorescent light (proportional to the nucleic acid content); WBC = white blood cell; nRBC = nucleated red blood cell. WDF color scheme: debris (dark blue), neutrophils (light blue), lymphocytes (pink), eosinophils (red), and monocytes (green). WNR color scheme: debris (dark blue), nRBCs (pink), WBCs (light blue), and basophils (yellow).

During this study period (August 2019 and June 2022), infrequent cases of misclassification of WBCs as nRBCs in the WNR channel were identified, which led to severe errors in the automated counts of both cell populations (marked normoblastosis and marked leukopenia). As an example, the case of Cat 51 is presented here. The associated WDF (WBC differential) and WNR scattergrams are shown in Figure 5, and the corresponding data are shown in Table 8. In contrast to the WNR channel, the WDF channel performs a white blood cell differential count using lysis and fluorescence staining procedures that leave the white blood cells largely intact for optimal cell differentiation. Cell populations are plotted according to their internal complexity/granularity (side scatter, SSC) and nucleic acid content (SFL) on the WDF scattergram [24,25].

The case of Cat 51 shows a tremendous underestimation of WBC counts (severe leukopenia; WBC count of 0.34 × 10^3^/µL; ref.: 4.6–12.8 × 10^3^/µL) and overestimation of nRBCs (severe normoblastosis; absolute nRBC count of 11.16 × 10^3^/µL, flagged with “@,” indicating a count outside the linearity limit), due to a misclassification of WBCs as nRBCs in the WNR channel. The manual nRBC counts revealed the absence of remarkable normoblastosis (0.5 nRBCs/100 WBCs), and manual re-gating of the WNR channel was performed accordingly (Figure 5B) to correct the error. Besides the severe error in WBC and nRBC counts, the WBC differential counts, especially the lymphocyte and neutrophil counts, differed severely before and after re-gating (Table 8), although the WDF scattergrams showed almost similar fractioning of cell populations (shown in the left graphs of Figure 5A,B) before and after re-gating. This finding was further investigated.

**Figure 6:**
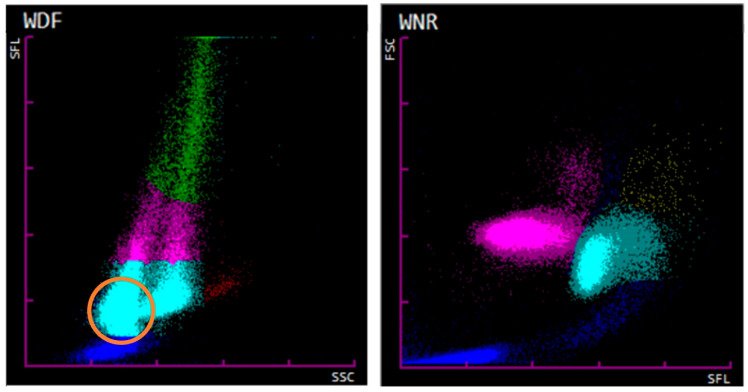
WDF (**left**) and WNR (**right**) scattergrams from a canine sample with severe normoblastosis. The WDF scattergram shows a poor distinction of cell populations and arbitrary fractioning of cells. Cells indicated in light blue (suspect neutrophils) are present as two populations. The left population (orange circle) represents the nucleated red blood cells (nRBCs) entering the WDF channel. The WNR scattergram shows the presence of severe normoblastosis (nRBCs shown in purple). Abbreviations: SSC = side scattered light (proportional to the granularity of the cell); FSC = forward scattered light (proportional to the cell size); SFL = side fluorescent light (proportional to the nucleic acid content). WDF color scheme: debris (dark blue), neutrophils (light blue), lymphocytes (pink), eosinophils (red), and monocytes (green). WNR color scheme: debris (dark blue), nRBCs (pink), leukocytes (light blue), and basophils (yellow).

Automated WBC counts by Sysmex XN-V are obtained from both channels (WDF and WNR channels) and drawn for comparison by performing a delta equation (Delta-WBC = WBC-D/WBC) as a quality control step. The optimum is a delta of 1 (equal WBC counts from the WDF and WNR channels). If the delta exceeds the preset limits, a “Review” action message will appear [26]. Despite the erroneous WBC count in the case of Cat 51, the WBC count demonstrated no flag or action message. The data of Cat 51 showed similar WBC counts in both channels, although the WDF scattergram showed the presence of a WBC population and the WNR scattergram showed the absence of a WBC population. This suggests that the WBC counts from the WDF channel were subsequently automatically corrected by the, in this case falsely, determined nRBC counts from the WNR channel within the Sysmex XN-V software (Version 3.03 (00-08)). Therefore, due to the correction of WBC counts from the WDF channel by the nRBC counts from the WNR channel, the delta became unreliable to indicate erroneous WBC counts, as seen in the case of Cat 51. Our findings highlight that evaluating the WBC count for a flag and the WDF scattergram alone did not point out the false WBC count. However, the WBC differential counts were flagged by “*” in the case of Cat 51, indicating low reliability of the data, which should initiate blood smear evaluation and manual WBC differential. The automatic correction of WBC counts in the WDF channel subsequently affected the lymphocyte and neutrophil counts in particular. The example in Figure 6 shows a dog specimen with a marked normoblastosis and the nRBCs entering the WDF channel affecting the WDF scattergram. Considering this, the automated correction might be reasonable in cases of true normoblastosis where nRBCs are falsely counted as leukocytes (mostly as lymphocytes or neutrophils) within the WDF channel. However, the correction of the WBC differential count may not be reliable, and in cases of normoblastosis, an interference of the nRBCs with the WBC differential must be considered. Therefore, a manual WBC differential should be initiated in cases with severe normoblastosis.

Our findings reinforce the importance of using a combination of non-statistical quality control procedures (inspection for flags, evaluation of all available scattergrams, and blood smear examination) to eliminate seldom but severe misclassifications of WBCs as nRBCs or the effect of nRBC counts on WBC and WBC differential counts.

## 4. Discussion

The performance of nRBC enumeration by the Sysmex XN series has been previously evaluated with human samples, revealing it to be a precise and accurate method, especially in specimens with less than 200 nRBCs/100 WBCs [27]. The overall concordance rate between the manual and automated counts by Sysmex XN has been shown to be high (85.1% and 93.2%) [16,27,28]. Previous studies on the veterinary analyzer Sysmex XN-V revealed a low imprecision with an overall CV of 7.1%. Additionally, good agreement has been found for feline and canine specimens with absolute nRBC counts of <1000 nRBCs/µL, excluding samples without circulating nRBCs in data analysis. In cases with higher nRBC counts, blood smear evaluation is recommended for confirmation due to a wider 95% LoA [29]. Correlation between the Sysmex XN-V and manual nRBC counts was found to be fair (*r* = 0.57) in a study using 63 canine specimens with a median automated absolute count of 10 nRBCs/µL (ranging from 0 to 1120 nRBCs/µL). This low correlation may be due to the low nRBC counts assessed and the poor reliability of the manual nRBC counting [30]. However, the performance of Sysmex XN-V in the enumeration of nRBCs using a wide range of feline and canine specimens, also including samples without normoblastosis, had not yet been evaluated. Therefore, the present method comparison study is unique in that it investigated a high number of patient samples at the full measurement range to detect both false negative and false positive results since they could affect patients’ treatment and outcome. In addition to previous studies, linearity, stability, carryover, and the investigation of interferences of nRBCs with the white blood cell differential in the WDF channel were studied.

The present study investigated a wide range of nRBC counts from 0 to 272 nRBCs/100 WBCs in dogs and 0 to 527 nRBCs/100 WBCs in cats, also including specimens without normoblastosis. A total of 3810 dog and 2844 cat specimens were used for assessing the agreement of the method. By analyzing patient samples collected in routine diagnostics over the course of three years, the study population reflects a representative patient population for veterinary laboratories. Since severe normoblastosis is an uncommon finding in canine and feline species, the majority of samples showed normal or low nRBC counts and samples with high numbers of nRBCs were less frequently observed. Still, in the group with >20 nRBCs/100 WBCs, 52 dog and 18 cat samples could be evaluated for this study. To overcome the limitation that the majority of evaluated specimens had normal or low nRBC counts, the performance of Sysmex XN-V was evaluated at three different severity levels of normoblastosis (low: <5, moderate: 5–20, and high: >20 nRBCs/100 WBCs) providing insight in the performance at different ranges. A fair to excellent correlation ranging from 0.743 to 0.998, with the highest correlation in the group with >20 nRBCs/100 WBCs in both species, was determined by regression analysis, indicating a representative study population and range.

The grading of relative or absolute nRBC counts is not harmonized in the veterinary literature. However, up to 1 nRBC/100 WBCs or absolute counts of <100 nRBCs/µL are most often considered normal. Nucleated RBCs of more than 5/100 WBCs or >400 nRBCs/µL are considered indicative of severe normoblastosis [4,31]. A relative count of >5/100 WBCs is also often used as the decision limit for the correction of WBC counts if no automatically corrected WBC count is available. Normoblastosis can be assessed as relative (number of nRBCs per 100 white blood cells) or absolute (number of nRBCs per microliter) counts. However, considering the increasing availability of automated nRBC counts in the future, medical decision limits should be set using absolute counts to eliminate the interference of WBC counts in the decision.

Manual nRBC counting was used as the reference method for method comparison, as it represents the most commonly used method for the enumeration of nRBCs in veterinary medicine. However, manual counting may miss low numbers of nRBCs and shows high imprecision and interobserver variation [16,17,20,29]. A high imprecision of manual counts was also noted in this study, with a mean CV of 19.6%. Manual imprecision is especially high at low ranges of nRBCs, as also previously reported [16,27]. This finding may be associated with the low numbers of cells counted manually, observer bias, and poor distribution of cells. Precision analysis of automated nRBC counts has shown low within- and between-run imprecision, with a mean CV of 4.5% and 5.4%, respectively. The imprecision of Sysmex XN-V determined in this study was found to be lower than previously reported [29]. Given the higher imprecision of manual counts compared to automated counts, data from method comparisons using manual counting as the reference method need to be interpreted in light of the possible unfavorable performance of the reference method (manual count). Method comparison of nRBC counts using a gold standard method, such as flow cytometric analysis and electron microscopy, has not yet been performed in veterinary medicine, possibly due to the low availability or unavailability of these methods. However, this gap should be filled in future investigations.

Stability testing showed no significant changes over 48 h at room temperature and refrigerated. Linearity was excellent at most investigated levels, especially at high levels of nRBC counts. However, at the lowest investigated level (<300 nRBC/µL), the difference exceeded the manufacturer’s specifications. A dilution error can be excluded since the linearity was excellent in red blood cell and white blood cell measurements in the investigated samples. According to our findings, linearity was confirmed at a range between 300 and 3000 nRBCs/µL. Carry-over was not evident. The limitations of this study include the relatively low number of samples used for the assessment of precision, linearity, and stability. This was due to the low numbers of specimens with increased nRBC counts available and the limited volume of residual samples, especially in cats.

Method comparison for the feline samples revealed a high overall concordance of 93%, with the highest concordance at the lowest ranges (≤1 nRBCs/100 WBCs). The mean bias was small at all levels. Although the 95% LoA was wider in the groups with moderate and high nRBC counts (>5 nRBCs/100 WBCs; 86 feline specimens), a normoblastosis was detected by Sysmex XN-V in all of these specimens. The clinical importance of wider 95% LoA at higher ranges is difficult to assess since no medical decision limits have been defined for nRBC counts in veterinary medicine. It therefore remains unclear if the observed variation would alter medical decisions. Furthermore, since severe normoblastosis was a rare finding in feline samples (3% samples with >5 nRBCs/100 WBCs), the overall agreement of the automated counts by Sysmex XN-V and the manual counts needs to be considered good in feline samples. However, manual counts may be performed to confirm the degree of normoblastosis in cases of high automated nRBC counts, since a wider variation is possible. If done so, the higher imprecision of manual counts must be considered when comparing the results.

Method comparison for the canine samples revealed a high overall concordance of 93%, with the highest concordance at the lowest ranges (≤1 nRBCs/100 WBCs). A negligible bias and narrow 95% LoA were found for specimens below 5 nRBCs/100 WBCs. A negative bias and wider 95% LoA were determined for samples with 5 or more nRBCs/100 WBCs, suggesting either an underestimation of automated counts or an overestimation of manual counts. Despite the better performance of manual counts at higher ranges, the imprecision of the manual counts still exceeded the very low imprecision of the automated counts. Therefore, the contribution of the poorer performance of the manual counts to the bias cannot be excluded. However, the negative proportional bias may be also driven by an underestimation of the automated counts in patients with severe normoblastosis. An underlying reason for this may be a poorer identification of early precursors (e.g., rubriblasts and prorubricytes), as previously hypothesized by Brown et al., where a negative proportional bias was also observed [29]. This hypothesis was not further investigated in the frame of the present study. In the canine specimens with manual relative nRBC counts above 5 (5.4% of all samples), normoblastosis was detected by Sysmex XN-V in all cases. The importance of wider 95% LoA in the canine specimens at higher nRBC counts must be correlated with medical decision limits, which are not yet defined for nRBC counts. Therefore, the influence of a wider 95% LoA and proportional negative bias on medical decisions remains unclear. However, given these findings, confirmation of nRBC counts by manual counts may be performed for samples with moderate and severe normoblastosis in addition to scattergram evaluation and inspection for flags.

The total observable error (TEobs) was below 9% for both species over the full range of investigation. No total allowable error (TEa) has yet been defined for the enumeration of nRBCs, and no expert opinion values are available to the best of the authors’ knowledge. Therefore, the TEobs was compared to the TEa for other common hematological values (TEa for WBC: 15%; TEa for RBC: 6–10%) defined by ASVCP QALS (American Society for Veterinary Clinical Pathology Quality Assurance and Laboratory Standards Committee) and CLIA (Clinical Laboratory Improvement Amendments) recommendations [32,33]. Thus, the TEobs for the enumeration of nRBCs by Sysmex XN-V was found to be within these limits, even for samples with marked normoblastosis, suggesting good performance of the assay investigated.

False classification of nRBCs, as shown by the example of Cat 51, probably exceeds medical decision limits for WBC counts and therefore will affect the treatment, test regimen, and, possibly, the outcome of the patient. However, this infrequently observed severe error was noticed by non-statistical quality control procedures, which were performed for each hematological evaluation according to a standard operating procedure, including inspection for flags, scattergram evaluation, and blood smear examination. If such a case was noticed, manual re-gating was performed after confirmation of the absence of a significant normoblastosis by blood smear evaluation. This procedure resulted in a good agreement between manual and automated nRBC counts and reliable values could be obtained. Brown et al. previously reported a correlation between a high or high normal hematocrit and the misclassification of WBC as nRBCs in the WNR channel [29]. This phenomenon can also be confirmed for our investigated cases including the case of Cat 51. A mean hematocrit of 54% in cats (ten cases) and a mean hematocrit of 56% in dogs (four cases) was found. Further investigation in cases with severe normoblastosis showed that WBC differentiation is also affected by nRBCs entering the WDF channel. This emphasizes the importance of manual WBC differentiation by blood smear evaluation in cases of remarkable normoblastosis. After the course of this study, Sysmex introduced the Sysmex XN-V Series IPU software update version 3.07-00 in July 2022 with improvements on the fractioning of nRBCs and WBCs in the WNR channel by adjusting the algorithm. Since then, significant misclassifications, as described by the case of Cat 51, were subjectively noted to a much lesser extent in our routine diagnostics. However, rare misclassifications of WBCs as nRBCs must still be anticipated, especially in patients showing erythrocytosis. Thus, non-statistical quality control procedures, such as scattergram evaluation, investigation for flags, and blood smear evaluation, should be carried out for patients with severe normoblastosis and concurrent leukopenia to identify possible errors.

Our findings suggest, for both species, that automated and manual nRBC counts can be used interchangeably in samples with low nRBC ranges (<5 nRBCs/100 WBCs). In the higher ranges, Sysmex XV-V performed satisfactorily compared to the manual method. Besides the essential evaluation for flags and scattergram examination, manual counts by blood smear evaluation are recommended for confirmation of the degree of normoblastosis in cases with high nRBC counts. The importance of blood smear evaluation in cases with remarkable normoblastosis is further supported by the observed interference of nRBCs with the WBC differentials and the rare chance of misclassification of WBCs as nRBCs.

The advantages of the automated enumeration of nRBCs include the readily and cost-effective availability of nRBC counts for each patient, the elimination of undetected significant normoblastosis, the reduced hands-on time, and increased precision of counts compared to manual counting. The elimination of undetected normoblastosis is especially important in patients with leukopenia and severe normoblastosis as, e.g., seen in oncological patients receiving chemotherapeutics (e.g., vincristine, doxorubicine, vinblastine, and prednisone), where drug-induced severe normoblastosis may mask concurrent leukopenia due to overestimation of automated leukocyte counts if no WBC correction is performed [4,19]. Furthermore, by using automated nRBC counts, this measurement will be readily available for more patients and can provide a foundation to propagate research on circulating nRBCs, providing further insight into, e.g., the regenerative response of anemic patients, the severity of disease, and the risk of mortality in critically ill patients.

## 5. Conclusions

The enumeration of nRBCs by Sysmex XN-V is an accurate and precise method. Sysmex XN-V automated nRBC counts can be used interchangeably with manual counts, although in patients with remarkable normoblastosis, counts should be confirmed by blood smear evaluation as part of a non-statistical quality control. This technological advancement will save time, increase precision, and eliminate undetected normoblastosis. Furthermore, the now readily available automated nRBC counts will add to future research regarding the significance of nRBCs in regenerative anemia and their value as a prognostic factor.

## Figures and Tables

**Table 1:** Imprecision of manual nucleated red blood cell counts (nRBCs/100 WBCs) by blood smear evaluation for the canine and feline specimens.

Material	Number of Counts	nRBCs/100 WBCs (Mean)	CV%
Canine M1	5	5.2	28.5
Canine M2	5	22	21.1
Canine M3	5	23	19.9
Canine M4	5	58	18.6
Feline M1	5	10	25.5
Feline M2	5	16	23.7
Feline M3	5	57	10.2
Feline M4	5	217	9.4

**Table 2:** Coefficients of variation (CVs) of the within-run imprecision (repeatability) and between-run imprecision (reproducibility) of the Sysmex XN-V hematological analyzer for the determination of absolute nRBC counts (nRBCs/µL) from the canine and feline specimens and quality control materials (Sysmex QC1–3).

Material	Number of Measurements	nRBCs/µL (Mean)	CV%
Repeatability			
Canine 1	5	330	7.4
Canine 2	5	960	3.1
Canine 3	5	2998	2.3
Feline 1	5	268	3.1
Feline 2	5	398	6.5
Reproducibility			
QC 1	20	148	7.9
QC 2	20	473	4.8
QC 3	20	1116	3.5

**Table 3:** Results of agreement comparing automated (Sysmex XN-V) and manually determined relative nRBC counts/100 WBCs of canine samples (*n* = 3810). Specimens were subdivided into three groups (<5; 5–20; >20 nRBCs/100 WBCs) according to their manually determined nRBC counts. The number of dogs (N) and means of the manually determined nRBC counts from each group are shown. The intercept and slope calculated by Deming regression analysis are shown with the 95% confidence interval in parentheses. Biases calculated by Bland–Altman difference analysis are shown with the 95% limits of agreement in parentheses. nRBCs = nucleated red blood cells; WBCs = white blood cells.

Group ^1^	N	Mean	Coefficient of Correlation (*r)*	Intercept *	Slope *	Bias ^+^
<5	3584	0.4	0.759	−0.10 (−0.14 to −0.07)	1.27 (1.16 to 1.38)	0.01 (−1.11 to 1.12)
5–20	174	9.4	0.754	−4.75 (−6.41 to −3.1)	1.37 (1.18 to 1.57)	−1.27 (−6.92 to 4.38)
>20–272	52	47	0.978	0.73 (−2.42 to 3.87)	0.82 (0.74 to 0.90)	−7.76 (−35.72 to 20.21)

^1^ Assigned by the manual count (nRBCs/100 WBCs). * With the 95% confidence interval in parentheses. ^+^ With the 95% limits of agreement in parentheses.

**Table 4:** Distribution of nucleated red blood cell (nRBC) counts from the manual method (nRBCm) and Sysmex XN-V (nRBCa) at four different ranges. Comparison of 3810 canine specimens. Concordant samples are highlighted in bold.

Range of nRBCs/100 WBCs by Manual Count (nRBCm)	Number of Samples (nRBCm)	Range of nRBCs/100 WBCs by Sysmex-XN-V (nRBCa) with Number of Samples			
		≤1	1.1–5	5.1–20	>20
**≤1**	3188	**3049**	139		
**1.1–5**	418	133	**263**	22	
**5.1–20**	152		26	**125**	1
**>20**	52			12	**40**

**Table 5:** Results of agreement comparing automated (Sysmex XN-V) and manual nRBC counts/100 WBCs of the feline samples (*n* = 2844). Specimens were subdivided into three groups (<5; 5–20; >20 nRBCs/100 WBCs) according to their manually determined nRBC counts. The number of cats (N) and means of the manually determined nRBC counts from each group are shown. The intercept and slope calculated by Deming regression analysis are shown with the 95% confidence interval in parentheses. Biases calculated by Bland–Altman difference analysis are shown with the 95% limits of agreement in parentheses. nRBCs = nucleated red blood cells; WBCs = white blood cells.

Group ^1^	N	Mean	Coefficient of Correlation (*r*)	Intercept *	Slope *	Bias ^+^
<5	2750	0.3	0.743	−0.04 (−0.07 to −0.017)	1.53 (1.4 to 1.66)	0.11 (−0.94 to 1.15)
5–20	76	10	0.759	−6.67 (−9.5 to −3.84)	1.66 (1.37 to 1.95)	−0.24 (−7.40 to 6.91)
>20–527	18	74	0.998	−4.28 (−9.54 to 0.97)	1.04 (0.98 to 1.10)	−1.31 (−18.46 to 15.83)

^1^ Assigned by the manual count (nRBCs/100 WBC). * With the 95% confidence interval in parentheses. ^+^ With the 95% limits of agreement in parentheses.

**Table 6:** Distribution of nucleated red blood cell (nRBC) counts from the manual method (nRBCm) and Sysmex XN-V (nRBCa) at four different ranges. Comparison of 2844 feline specimens. Concordant samples are highlighted in bold.

Range of nRBCs/100 WBCs by Manual Count (nRBCm)	Number of Samples (nRBCm)	Range of nRBCs/100 WBCs by Sysmex-XN-V (nRBCa) with Number of Samples			
		≤1	1.1–5	5.1–20	>20
**≤1**	2550	**2421**	128	1	
**1.1–5**	208	47	**152**	9	
**5.1–20**	68		13	**49**	6
**>20**	18			4	**14**

**Table 7:** Changes (%) of absolute nRBC counts in dog and cat samples stored at either room temperature (RT) or 4 °C for 48 h. Absolute nRBC counts were determined by Sysmex XN-V. The direction of change is shown by the arrows next to the percentage. N.A. = not available; ↓ = decrease; ↑ = increase.

**Sample and** **Storage Temperature**	**nRBC/µL**	**4 h**	**8 h**	**24 h**	**48 h**
Dog (RT)	1380	4.4% ↓	3.6% ↓	2.9% ↓	2.2% ↓
Dog (4 °C)	2360	4.7% ↑	7.6% ↑	7.2% ↑	7.2% ↑
Cat (RT)	360	8.3% ↓	0.0%	2.8% ↑	N.A.
Cat (4 °C)	2440	2.0% ↑	0.4% ↑	3.7% ↑	12.7% ↑

**Table 8:** Automated Sysmex XN-V measurements from a feline EDTA-anticoagulated whole blood specimen (Cat 51) presenting a misclassification of WBCs as nRBCs in the WNR channel. Cell counts are shown before and after manual re-gating. Flags are indicated for each measurement if present. The corresponding WDF and WNR scattergrams are shown in Figure 5A,B.

Measurand	Before Re-Gating (Figure 5A)		After Manual Re-Gating (Figure 5B)	
	Count	Flag	Count	Flag
WBCs (10^3^/µL)	0.34		11.46	
nRBC# (10^3^/µL)	11.16		0.00	
nRBCa (/100 WBCs)	3282	@	0.0	
Neutrophils (10^3^/µL)	0.00	*	8.08	
Lymphocytes (10^3^/µL)	0.00	*	2.68	
Monocytes (10^3^/µL)	0.04	*	0.26	
Eosinophils (10^3^/µL)	0.29	*	0.46	
Basophils (10^3^/µL)	0.01	*	0.01	

* Low-reliability flag; this indicates that the reliability of the data is low. @ Out-of-range flag; this indicates that the data are outside the linearity limit.

## Data Availability

All available data are presented in this manuscript.
